# The Association of Resistin with Metabolic Health and Obesity in a Mexican-American Population

**DOI:** 10.3390/ijms26094443

**Published:** 2025-05-07

**Authors:** Reem Al-Dallal, Keziah Thomas, MinJae Lee, Aysha Chaudhri, Eleanor Davis, Priyanka Vaidya, Miryoung Lee, Joseph B. McCormick, Susan P. Fisher-Hoch, Absalon D. Gutierrez

**Affiliations:** 1 Department of Internal Medicine, Division of Endocrinology, Diabetes and Metabolism, The University of Texas Health Science Center, Houston, TX 77030, USAkeziah.m.thomas@uth.tmc.edu (K.T.);; 2 Department of Health Data Science & Biostatistics, Peter O’Donnell Jr. School of Public Health, University of Texas Southwestern, Dallas, TX 75390, USA; minjae.lee@utsouthwestern.edu; 3 Department of Endocrine Neoplasia and Hormonal Disorders, Division of Internal Medicine, The University of Texas MD Anderson Cancer Center, Houston, TX 77030, USA; 4 Department of Epidemiology, Human Genetics and Environmental Sciences, The University of Texas Health Science Center at Houston School of Public Health, Brownsville Regional Campus, Brownsville, TX 78520, USA; miryoung.lee@uth.tmc.edu (M.L.);

**Keywords:** resistin, leptin, adiponectin, Mexican-American, metabolic health, obesity, IL-1β, IL-6, TNF-α, IL-8

## Abstract

Research on the relationship between resistin levels, metabolic health, and obesity has produced inconsistent findings across different ethnic groups, making it unclear whether lower resistin levels are associated with these conditions in Mexican-Americans. This cross-sectional study investigated the relationship between resistin, metabolic health, and obesity in an adult Mexican-American cohort (*n* = 1511) using multivariable linear regression analysis. Related adipokines (leptin and adiponectin) were measured simultaneously. Participants were categorized into four groups by metabolic health (healthy/unhealthy) and obesity (obese/non-obese) status. “Metabolically unhealthy” was defined as ≥2 cardiometabolic abnormalities. Obesity was defined as a BMI ≥ 30 kg/m^2^. We also investigated the associations of related proinflammatory cytokines, demographic/anthropometric variables, and medications with each outcome variable of interest. The results showed no statistically significant differences in resistin levels between the groups. Leptin was higher and adiponectin was lower in groups with obesity and/or metabolically unhealthy status. The resistin findings contrast studies in other populations, while other leptin and adiponectin findings confirm those seen in many ethnic groups. Thiazolidinedione use was associated with lower resistin, confirming earlier research. These findings suggest that resistin’s role in metabolic health may be different in Mexican-Americans compared to other populations.

## 1. Introduction

Although lower levels of circulating resistin improve insulin sensitivity (IS) and metabolic health in obese mice [1], it is uncertain whether this phenomenon also applies to humans, due to conflicting results from population studies [2,3,4,5,6]. Notably, resistin is secreted primarily by adipocytes in mice [1] and primarily by macrophages (and secondarily by adipocytes) in humans [7,8,9]. However, as adipose tissue (AT) is infiltrated by macrophages in obesity, resistin might remain a relevant factor in obesity-related metabolic health.

Studies in Europeans show that higher circulating resistin is associated with increased cardiovascular death rates, cardiovascular disease (CVD), and all-cause mortality in metabolically diverse individuals [10,11,12,13]. A putative mechanism suggests that resistin regulates specific proinflammatory cytokines (IL-1β, IL-6, IL-8, and TNF-α) to create an inflammatory cascade facilitating CVD [11].

It is unclear if the above findings translate to Mexican-Americans. Our prior work in this group demonstrated lower circulating resistin associating with decreased IS [14]. Similarly, higher circulating resistin is associated with lower CVD risk in subjects with diabetes mellitus [15]. Given the paucity of studies and conflicting findings with Europeans, one must better define the effects of resistin in Mexican-Americans.

The primary objective of this study is to characterize the relationship between circulating resistin levels and metabolic health and obesity in a Mexican-American cohort. Concurrently, we assess how resistin levels are affected by the aforementioned signaling cytokines (IL-1β, IL-6, TNF-α, and IL-8), as well as demographic and anthropometric variables. A comparative analysis of the relationships between metabolic health/obesity and leptin and adiponectin was conducted using the same assessments.

## 2. Results

### 2.1. Descriptive Demographic, Anthropometric, and Metabolic Characteristics by Metabolic Health and BMI Category

The baseline analyses were performed on 1511 Mexican-American subjects (42.0% male, average age of 44.75 ± 15.20 years, average BMI of 31.01 ± 6.34 kg/m^2^). Means and standard deviations (SDs) are reported for normally distributed continuous data; medians and interquartile ranges (IQRs) are reported for non-normally distributed data in Table 1. As expected, hemoglobin A1c, insulin, HOMA-IR, triglyceride, and total values were significantly higher in the MHO, MUHNW, and MUHO groups. HDL-C levels were lower in the MUHNW and MUHO groups, and there were no statistically significant differences in LDL-C levels between groups. Leptin levels were higher among obese groups (MHO and MUHO) while adiponectin levels were higher among metabolically healthy groups (MHNW and MHO). The distribution of resistin data was not statistically different between the four categories. Levels of IL-6, TNF-α, and IL-8 were lower among MHNW subjects, while IL-1β showed no difference between groups.

### 2.2. Univariable and Multivariable Associations Between Metabolic Health and BMI with Adipokines

We assessed both univariable and multivariable associations of metabolic health and BMI (defined by four groups, i.e., MHNW, MHO, MUHNW, MUHO) with log-transformed values of adipokines (resistin, leptin, and adiponectin). The unadjusted (Model 1) and adjusted (Models 2 and 3) mean differences in log-transformed resistin (Table 2), leptin (Table 3), and adiponectin (Table 4) between groups are presented. Table S1 shows further adjustments for each medication (statins, TZDs, incretin mimetics, ACEIs/ARBs, fibrates, or NSAIDs) in each adipokines model. Of note, markers of inflammation in Models 2–4 were selected to reflect similar models from Menzaghi et al., who described a resistin–cytokine pathway in humans [11].

The natural log of resistin levels (Table 2) showed no statistically significant differences between groups in Model 1 (all *p* > 0.1). Model 2 adjustments showed similar findings, but with small, statistically significant associations by IL-8 and TNF-α (*p* < 0.05 for each). Further, Model 3 adjustments showed the same small associations by both cytokines (*p* < 0.05 for each). The use of thiazolidinediones (TZDs), which were primarily by subjects in the MUHNW and MUHO groups (Table 1), was associated with clinically and statistically significant lowering of resistin (mean decrease of 0.862 ng/mL, *p* = 0.0005) (Table S1).

The natural log of leptin levels (Table 3) was significantly higher among groups including obese and metabolically unhealthy individuals versus their nonobese and metabolically healthy counterparts in Model 1 (*p* < 0.0001 for these comparisons). Levels were lower among MUHNW versus MHO (*p* < 0.0001), and no statistically significant differences were noted between MUHO vs. MHO. Model 2 showed similar findings along with an additional small, positive association with IL-8 (*p* = 0.0174). Model 3 showed similar findings between groups, minus the aforementioned IL-8 association, and with the additions of a small, positive association with TNF-α (*p* = 0.046) and a larger negative association with male sex (mean decrease of −1.123 ng/mL, *p* < 0.0001). Fibrates were associated with reduced leptin (mean decrease of 0.287 ng/mL, *p* = 0.0071) (Table S1).

The natural log of adiponectin levels (Table 4) was significantly lower among groups including obese and metabolically unhealthy individuals versus their nonobese and metabolically healthy counterparts in Model 1 (*p* < 0.05 for these comparisons). There were no statistically significant differences between MUHNW versus MHO. Model 2 further showed small increases associated with IL-6 (*p* = 0.0425) and IL-8 (*p* = 0.0002). These trends persisted in Model 3, with an additional small increase with age (*p* = 0.0001) and a moderate reduction with male sex (mean reduction of 0.259 µg/mL, *p* < 0.0001). Fibrates were associated with reduced adiponectin (mean decrease of 0.169 µg/mL, *p* = 0.0402) (Table S1).

## 3. Discussion

Among this cohort of Mexican-American subjects, circulating resistin levels did not differ significantly between groups with varying metabolic health or obesity statuses. Leptin and adiponectin levels, however, aligned with previously expected patterns for metabolic health and obesity in diverse populations.

Prior research has yielded conflicting results regarding the link between resistin and metabolic health/obesity. While some studies conducted in Mexico, the United States, and Japan have reported higher resistin levels in obese individuals compared to their non-obese counterparts [16,17,18], others, primarily in Europe and the United States, have found no such association [19,20,21]. Some European studies uncovered a strong association between higher resistin levels and metabolic syndrome components [3,22], but this finding was not replicated in a US-based cohort [23]. Unlike prior studies suggesting a multi-cytokine pathway (involving IL-1β, IL-6, IL-8, and TNF-α) targeting resistin [11,12], there were no detectable effects from IL-1β or IL-6 and the effects of IL-8 and TNF-α were very small. Inconsistent results may be explained by different population ethnicities, study sample sizes, other diseases present, tissues analyzed, and variations in resistin assays [24]. One may also reasonably speculate that genetic variance influenced our findings, as various single nucleotide polymorphisms (SNPs) are shown to impact resistin levels in Mestizo-Mexican and Northeastern US populations [5,25]. However, TZDs (used by a small number of subjects; Table 1) were associated with statistically significant lower levels of resistin, which is consistent with prior literature across multiple ethnicities [26,27,28].

Compared to all normal-weight and metabolically healthy individuals, leptin levels were higher among those with obesity and/or metabolically unhealthy status, with a larger effect observed in obesity. Numerous studies have established the association of increased leptin with increased obesity in Mexican populations [29,30]. In both obese and non-obese individuals from European and US populations, leptin levels correlated positively with the metabolic syndrome [31,32,33]. While other research on the relationship between leptin and metabolic syndrome in Mexican-Americans is limited, some studies suggest a negative association with insulin resistance [14,34], consistent with findings in European and Middle Eastern populations [35,36,37]. In our data, MHO subjects also displayed higher leptin levels than MUHNW subjects, suggesting a dominant role for obesity. As shown previously, females displayed higher leptin levels (Model 3) [38]. The effects of IL-8 and TNF-α were very small. Notably, some genetic studies show that SNPs of LEP and LEPR genes may affect leptin levels in Mexicans [39,40]. Fibrate therapy (used by a small number of subjects; Table 1) was associated with the expected reduction in leptin [41].

Lower adiponectin levels were observed in groups with obesity and/or metabolically unhealthy status (with a larger effect in metabolically unhealthy status), compared to their healthy and metabolically healthy counterparts. The obesity finding, along with higher levels in females, is consistent with a prior study in Mexican-Americans [29]. Adiponectin levels were lower in the metabolic syndrome, particularly among women, as noted in both obese and non-obese European and US subjects [31,32,33]. The effects of IL-6, IL-8, and age were statistically significant but very small. Fibrate therapy was associated with the expected reduction in adiponectin [41]. Surprisingly, TZDs were not associated with a statistically significant increase in adiponectin [42]. However, adiponectin levels (in TZD-treated subjects) were higher in female sex, showing positive associations with age and negative associations with obesity (Supplementary Table S1); this is consistent with prior findings in Mexican-Americans [29]. A positive association between adiponectin and IL-8 level coincides with evidence that adiponectin increases IL-8 in some inflammatory diseases [43]. As Mexican-Americans reflect a diverse multiethnic group [44,45], it is imperative to study these individuals from a genetic perspective.

Several limitations should be acknowledged. The cross-sectional nature of the study design means we can only observe associations and cannot infer cause-and-effect relationships. We are also unable to determine the specific temporal dynamics between the variables. Additionally, the study population consisted solely of Mexican-Americans, which restricts the extent to which our results can be generalized to other Hispanic/Latino subgroups or individuals of different ethnicities. Differences in genetic variability may have played a role in our findings, given the high prevalence of Amerindian genes in this cohort [44].

## 4. Materials and Methods

This cross-sectional study was approved by The Committee for the Protection of Human Subjects at The University of Texas Health Science Center at Houston. All procedures were performed in accordance with the Declaration of Helsinki of 1975.

### 4.1. Study Participants

Study participants were selected from the Cameron County Hispanic Cohort (CCHC), an ongoing randomly ascertained community-based cohort study recruited from households in a homogeneous Mexican-American population living along the southern Texas border. Cohort participants were enrolled with written informed consent, followed by the questionnaire completion (addressing anthropometric and socio-demographic characteristics), clinical examinations, and laboratory specimen collections as previously described [46].

Only adult subjects with resistin, leptin, and adiponectin levels, as well as all relevant study measures, were included in the study. Participants with hypo- or hyperthyroidism, major cardiovascular events, or active malignancy were excluded. At the time of this analysis, the cohort comprised 5038 subjects (recruited between 2004 and 2022). Of these, 4603 subjects were adults (18 years or older). Of these adults, 1511 met the inclusion criteria.

### 4.2. Definitions

Based on metabolic health and body mass index (BMI), participants were stratified into the following four categories, based on prior criteria [47,48]: (a) Metabolically Healthy Normal Weight (MHNW), (b) Metabolically Healthy Obese (MHO), (c) Metabolically Unhealthy Normal Weight (MUHNW), or (d) Metabolically Unhealthy Obese (MUHO).

Metabolic health was defined based on the participant’s blood pressure, fasting blood glucose, triglyceride and HDL levels, HOMA-IR value, and hsCRP level. A participant is considered metabolically unhealthy if two or more of the following are present: systolic blood pressure ≥ 130 mmHg and/or DBP ≥ 85 mmHg or on antihypertensive medications; triglyceride level ≥ 150 mg/dL, HDL-C level < 40 mg/dL in men or <50 mg/dL in women fasting blood glucose ≥ 100 mg/dL or taking hypoglycemic medications or homeostasis model assessment of insulin resistance (HOMA-IR) value of >5.13; or high sensitivity C-reactive protein of >3 mg/L [47,48]. Based on BMI, participants were categorized as either obese (BMI ≥ 30 kg/m^2^) or normal weight (BMI is <30 kg/m^2^).

### 4.3. Laboratory Measurements

Fasting blood specimens were immediately aliquoted and stored at −80 °C until analyzed. Measurements for fasting blood glucose, glycated hemoglobin (HbA1c), lipids, and insulin were conducted as previously described [14,19]. HOMA-IR was calculated using the following formula: fasting plasma insulin (FPI) multiplied by fasting blood glucose (FBG) in mmol/L, divided by 22.5. HOMA B (HOMA%Beta) was calculated using the following formula: 20 multiplied by FPI, divided by fasting plasma glucose (FPG) minus 3.5 [49]. In addition, high-sensitivity C-reactive protein (hsCRP) levels were measured using a high-sensitivity immunoassay (Dimension Vista 1500; Siemens Corporation, Washington, DC, USA). hsCRP levels >10 mg/L were excluded from analysis as such high levels likely represent acute illness [47].

Resistin, leptin, total adiponectin, and four relevant inflammatory markers (interleukin-1 beta (IL-1β), interleukin-6 (IL-6), interleukin-8 (IL-8), and tumor necrosis factor-alpha (TNF-α)) were measured using the multiplex ELISA (Milliplex Map, Millipore, CA, USA) bead technique as previously described [34]. The results were read using the Luminex 200 System (Luminex Corp, Austin, TX, USA).

### 4.4. Statistical Analysis

We first conducted univariable comparisons of demographic, anthropometric, and metabolic characteristics for the study participants grouped by their metabolic health status and BMI category using the Chi-square test for categorical variables, ANOVA, or their nonparametric equivalents (Kruskal–Wallis test) as appropriate for continuous variables. Due to the resistin assay’s lower limit of detection (LOD), 18.77% (*n* = 331) of left-censored data were imputed using the LOD (i.e., 1.28). We performed several sensitivity analyses, including the exclusion of censored values (using an *n* = 1180), as well as imputing them using either half the LOD or the LOD itself with the full sample (*n* = 1511). Our primary findings remained consistent and robust across these different approaches. As distributions of resistin, leptin, and adiponectin were skewed, the data were transformed using the natural logarithm (ln) to produce approximately normal distributions for the main statistical analysis. We examined the relationship of metabolic health and BMI with adipokines using multivariable linear regression models. Model 1 examined the unadjusted relationship of metabolic health and BMI with adipokines. Model 2 adjusted for markers of inflammation (IL-1β, IL-6, TNF-α, and IL-8). Model 3 additionally adjusted for demographic and anthropometric characteristics (sex, age, and smoking status), while Model 4 further adjusted for the use of medications which would potentially affect adipokine levels (statins, thiazolidinediones, incretin mimetics (GLP-1 receptor agonists and DPP4 inhibitors), angiotensin-converting enzyme inhibitors (ACE-Is)/angiotensin receptor blockers (ARBs), fibrates, and non-steroidal anti-inflammatory drugs (NSAIDs)). We also evaluated underlying assumptions such as normality and linearity, while building multivariable linear regression models. Multicollinearity among the adjusted variables in relation to each outcome variable was also assessed; all analyses were performed on SAS^®^ v. 9.4 (SAS Institute Inc., Cary, NC, USA) with a significance level of 0.05, using the comparisons described above.

## 5. Conclusions

**Figure 1:**
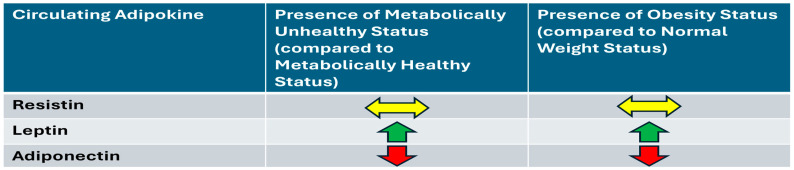
Overall associations between circulating adipokines and metabolic health/weight status in fully adjusted model (Model 3).

In this homogenous Mexican-American cohort, circulating resistin levels were not significantly different between groups with varying metabolic health or obesity statuses. This contrasts with findings in other populations. However, consistent with observations in many other populations, TZD uses were associated with lower resistin [26,27,28]. Unlike findings in European subjects, this study did not replicate previous observations of a resistin-involved pro-inflammatory cytokine pathway [11,12]. Leptin and adiponectin levels behaved as expected across the different study groups, consistent with patterns observed in other ethnicities. The overall findings of the study are illustrated in Figure 1. Future studies should investigate genetic variations within this population, particularly single nucleotide polymorphisms (SNPs), which differentially influence amounts of resistin and insulin resistance between Mexican, Chinese, and European populations [25,50,51]. Indeed, the influence of resistin on metabolic health and obesity might vary among Mexican-Americans compared to other groups.

## Figures and Tables

**Table 1:** Demographic, anthropometric and metabolic characteristics by metabolic health and obesity status.

Variable (Continuous)	ALL *n* = 1511	Metabolically Healthy Normal Weight (MHNW) *n* = 362 (23.96%)	Metabolically Healthy Obese (MHO) *n* = 160 (10.59%)	Metabolically Unhealthy Normal Weight (MUHNW) *n* = 361 (23.89%)	Metabolically Unhealthy Obese (MUHO) *n* = 628 (41.56%)	*p*-Value *
**Sex,** male *n* (%)	635 (42.03)	161 (44.48)	55 (34.38)	163 (45.15)	256 (40.76)	0.0860
**Age (years)**, mean (SD)	44.75 (15.20)	38.15 (14.87)	40.21 (13.15)	48.91 (15.70)	47.33 (14.04)	<0.0001
**BMI (kg/m^2^)**, mean (SD)	31.01 (6.34)	25.25 (2.82)	35.11 (5.25)	26.90 (2.44)	35.61 (5.21)	<0.0001
**WAIST CIRCUMFERENCE (cm)**, mean (SD)	102.9 (14.90)	89.39 (8.71)	108.9 (11.83)	95.82 (8.41)	113.2 (12.82)	<0.0001
**HbA1c (%)**, median (IQR)	5.45 (4.67, 6.20)	5.00 (4.19, 5.70)	5.20 (4.50, 5.80)	5.60 (4.84, 6.40)	5.72 (4.81, 6.79)	<0.0001
**Fasting Blood Glucose mg/dL**, median (IQR)	98.00 (92.00, 109.0)	92.50 (88.00, 96.00)	93.00 (88.00, 98.00)	102.0 (95.00, 114.0)	105.0 (97.00, 126.0)	<0.0001
**Insulin (pmol/L)**, median (IQR)	12.10 (7.70, 18.80)	8.10 (5.40, 12.20)	12.00 (7.50, 15.70)	11.15 (7.70, 16.30)	16.80 (10.50, 25.80)	<0.0001
**HOMA-IR**, median (IQR)	3.10 (1.93, 5.11)	1.82 (1.19, 2.81)	2.80 (1.70, 3.76)	3.01 (1.99, 4.77)	4.81 (3.00, 7.63)	<0.0001
**HOMA B**, median (IQR)	117.8 (73.01, 185.1)	99.31 (70.00, 157.2)	138.2 (96.92, 209.3)	100.4 (62.53, 156.0)	137.3 (76.68, 224.2)	<0.0001
Resistin (ng/mL) **, median (IQR)	18.87 (9.96, 28.96)	17.76 (9.01, 25.18)	18.97 (8.71, 30.84)	19.08 (8.40, 30.44)	19.53 (11.59, 29.85)	0.1005
**Leptin (ng/mL)**, median (IQR)	16.76 (7.28, 29.19)	7.98 (3.49, 17.50)	27.12 (13.59, 41.26)	11.13 (5.12, 20.86)	23.45 (13.73, 36.92)	<0.0001
**Adiponectin (µg/mL)**, median (IQR)	14.35 (9.74, 20.47)	17.16 (11.87, 24.71)	15.34 (10.95, 21.38)	14.67 (10.36, 20.73)	12.48 (8.52, 18.14)	<0.0001
**Triglycerides_mg/dL**, median (IQR)	129.0 (89.00, 185.5)	90.00 (65.00, 119.0)	101.0 (73.00, 129.0)	154.0 (104.0, 211.5)	156.0 (116.0, 219.0)	<0.0001
**Total Cholesterol_mg/dL**, mean (SD)	182.5 (38.90)	178.4 (39.37)	180.6 (35.42)	183.4 (39.27)	184.8 (39.13)	0.0416
**HDL-C_mg/dL**, mean (SD)	45.86 (11.46)	51.85 (11.95)	50.61 (10.34)	43.60 (10.60)	42.48 (10.06)	<0.0001
**LDL-C_mg/dL**, mean (SD)	107.8 (33.49)	107.8 (34.16)	108.2 (28.98)	107.7 (35.51)	107.8 (33.04)	0.8902
**IL-1β (pg/mL)**, median (IQR)	0.64 (0.49, 1.20)	0.64 (0.45, 1.20)	0.64 (0.49, 1.15)	0.64 (0.51, 1.20)	0.64 (0.51, 1.20)	0.6647
**IL-6 (pg/mL)**, median (IQR)	2.17 (0.95, 4.38)	1.22 (0.64, 2.48)	2.39 (0.73, 4.91)	2.31 (0.95, 4.29)	2.83 (1.51, 5.05)	<0.0001
**TNF-α (pg/mL)**, median (IQR)	2.17 (0.95, 4.38)	1.22 (0.64, 2.48)	2.39 (0.73, 4.91)	2.31 (0.95, 4.29)	2.83 (1.51, 5.05)	<0.0001
**IL-8 (pg/mL)**, median (IQR)	4.56 (3.36, 6.40)	4.13 (3.14, 5.55)	4.41 (3.33, 5.94)	4.67 (3.51, 6.75)	4.80 (3.45, 6.75)	<0.0001
**Hypertension (yes)**, *n* (%)	416 (28.09)	17 (4.71)	16 (10.26)	129 (36.24)	254 (41.78)	<0.0001
**Smoking History**, *n* (%)	488 (33.15)	107 (29.64)	39 (25.16)	131 (37.22)	211 (34.93)	0.0189
**Statins**, *n* (%)	265 (17.55)	30 (8.29)	7 (4.38)	79 (21.88)	149 (23.76)	<0.0001
**TZDs**, *n* (%)	26 (1.72)	1 (0.28)	0 (0.00)	10 (2.77)	15 (2.39)	0.0103
**Incretin mimetics**, *n* (%)	45 (2.98)	1 (0.28)	0 (0.00)	14 (3.88)	30 (4.78)	<0.0001
**ACEIs/ARBs**, *n* (%)	340 (22.52)	10 (2.76)	9 (5.63)	104 (28.81)	217 (34.61)	<0.0001
**Fibrates**, *n* (%)	43 (2.85)	1 (0.28)	2 (1.25)	13 (3.60)	27 (4.31)	0.0013
**NSAIDS**, *n* (%)	244 (16.16)	32 (8.84)	13 (8.13)	67 (18.56)	132 (21.05)	<0.0001

* *p*-values from Kruskal-Wallis test for non-normal continuous variables (summary statistics reported as median and interquartile range (IQR)), ANOVA for normal continuous variables (summary statistics reported as mean and standard deviation (SD)). *p*-values from Chi-square test for categorical variables. ** Due to the lower limit of detection, 18.77% of resistin data (*n* = 331) were imputed by the detection limit.

**Table 2:** Associations between metabolic health/obesity status and log-transformed resistin based on univariable (unadjusted) and multivariable (adjusted) linear regression models.

Ln Resistin	Model 1 (Unadjusted)		Model 2 (Adjusted)		Model 3 (Adjusted)	
	Mean Difference (95% CI)	*p*-Value	Mean Difference (95% CI)	*p*-Value	Mean Difference (95% CI)	*p*-Value
**Metabolic Health/Obesity Group**		0.4888		0.7294		0.8252
MHO vs. MHNW	0.090 (−0.149, 0.329)	0.4588	0.089 (−0.142, 0.320)	0.4488	0.084 (−0.151, 0.319)	0.4817
MUHNW vs. MHNW	0.066 (−0.121, 0.253)	0.4890	0.035 (−0.146, 0.216)	0.7032	0.033 (−0.155, 0.222)	0.7311
MUHO vs. MHNW	0.130 (−0.036, 0.296)	0.1236	0.085 (−0.075, 0.246)	0.2978	0.071 (−0.096, 0.238)	0.4054
0.4054	−0.024 (−0.263, 0.215)	0.8425	−0.054 (−0.285, 0.177)	0.6468	−0.051 (−0.291, 0.188)	0.6745
MUHO vs. MHO	0.040 (−0.183, 0.263)	0.7239	−0.004 (−0.220, 0.212)	0.9721	−0.013 (−0.236, 0.209)	0.9061
MUHO vs. MUHNW	0.064 (−0.102, 0.230)	0.4477	0.050 (−0.110, 0.210)	0.5390	0.038 (−0.126, 0.201)	0.6499
IL-1Beta pg/mL			−0.006 (−0.015, 0.003)	0.1936	−0.010 (−0.021, 0.001)	0.0690
IL-6_pg/mL			−0.0001 (−0.001, 0.0001)	0.7609	−0.0001 (−0.001, 0.0001)	0.8529
TNF-Alfa pg/mL			−0.010 (−0.020, −0.001)	0.0288	−0.011 (−0.020, −0.002)	0.0205
IL-8 pg/mL			0.076 (0.063, 0.090)	<0.001	0.077 (0.063, 0.091)	<0.001
Sex: Male vs. Female					0.040 (−0.099, 0.179)	0.5732
Age (year)					−0.003 (−0.007, 0.002)	0.2370
Smoking History: yes vs. no					0.117 (−0.028, 0.262)	0.1141

**Table 3:** Associations Between Metabolic Health/Obesity Status and Log-Transformed Leptin based on Univariable (Unadjusted) and Multivariable (Adjusted) linear regression models.

Ln Leptin	Model 1 (Unadjusted)		Model 2 (Adjusted)		Model 3 (Adjusted)	
	Mean Difference (95% CI)	*p*-Value	Mean Difference (95% CI)	*p*-Value	Mean Difference (95% CI)	*p*-Value
**Metabolic Health/Obesity Group**		<0.001		<0.001		<0.001
MHO vs. MHNW	1.139 (0.975, 1.303)	<0.001	1.140 (0.976, 1.304)	<0.001	1.026 (0.898, 1.154)	<0.001
MUHNW vs. MHNW	0.303 (0.174, 0.431)	<0.001	0.291 (0.163, 0.420)	<0.001	0.289 (0.187, 0.392)	<0.001
MUHO vs. MHNW	1.091 (0.977, 1.205)	<0.001	1.071 (0.956, 1.185)	<0.001	1.034 (0.943, 1.125)	<0.001
MUHNW vs. MHO	−0.836 (−1.000, −0.672)	<0.001	−0.849 (−1.013, −0.685)	<0.001	−0.737 (−0.866, −0.607)	<0.001
MUHO vs. MHO	−0.048 (−0.201, 0.105)	0.5361	−0.070 (−0.223, 0.084)	0.3736	0.008 (−0.113, 0.129)	0.8935
MUHO vs. MUHNW	0.788 (0.674, 0.902)	<0.001	0.779 (0.665, 0.893)	<0.001	0.745 (0.656, 0.834)	<0.001
IL-1Beta pg/mL			0.004 (−0.002, 0.011)	0.1711	0.005 (−0.001, 0.010)	0.1195
IL-6 pg/mL			−0.0001 (−0.001, 0.0001)	0.2269	−0.0002 (−0.0005, 0.0001)	0.2987
TNF-Alfa pg/mL			0.002 (−0.005, 0.008)	0.5718	0.005 (0.0001, 0.010)	0.0460
IL-8 pg/mL			0.012 (0.002, 0.021)	0.0174	0.006 (−0.002, 0.013)	0.1448
Sex: Male vs. Female					−1.123 (−1.199, −1.048)	<0.001
Age (year)					−0.0001(−0.003, 0.002)	0.8126
Smoking History: yes vs. no					−0.064 (−0.143, 0.015)	0.1133

**Table 4:** Associations between metabolic health/obesity status and log-transformed adiponectin based on univariable (unadjusted) and multivariable (adjusted) linear regression models.

Ln Adiponectin	Model 1		Model 2		Model 3	
	Mean Difference (95% CI)	*p*-Value	Mean Difference (95% CI)	*p*-Value	Mean Difference (95% CI)	*p*-Value
**Metabolic Health/Obesity Group**		<0.001		<0.001		<0.001
MHO vs. MHNW	−0.133 (−0.237, −0.028)	0.0130	−0.145 (−0.250, −0.041)	0.0065	−0.178 (−0.276, −0.080)	<0.001
MUHNW vs. MHNW	−0.202 (−0.285, −0.120)	<0.001	−0.212 (−0.294, −0.130)	<0.001	−0.309 (−0.388, −0.230)	<0.001
MUHO vs. MHNW	−0.332 (−0.404, −0.259)	<0.001	−0.343 (−0.416, −0.271)	<0.001	−0.446 (−0.516, −0.376)	<0.001
MUHNW vs. MHO	−0.070 (−0.175, 0.035)	0.1912	−0.066 (−0.171, 0.039)	0.2150	−0.131 (−0.231, −0.031)	0.0104
MUHO vs. MHO	−0.199 (−0.297, −0.101)	<0.001	−0.198 (−0.296, −0.100)	<0.001	−0.268 (−0.362, −0.175)	<0.001
MUHO vs. MUHNW	−0.129 (−0.202, −0.056)	<0.001	−0.132 (−0.204, −0.059)	<0.001	−0.137 (−0.206, −0.069)	<0.001
IL-1Beta pg/mL			−0.002 (−0.006, 0.002)	0.2986	−0.004 (−0.008, 0.001)	0.1070
IL-6 pg/mL			0.0002 (0.0001, 0.0005)	0.0425	0.0002 (0.0001, 0.0005)	0.0405
TNF-Alfa pg/mL			0.0001 (−0.004, 0.005)	0.8325	0.0001 (−0.004, 0.004)	0.9304
IL-8 pg/mL			0.012 (0.005, 0.018)	<0.001	0.010 (0.004, 0.016)	0.0010
Sex: Male vs. Female					−0.259 (−0.317, −0.201)	<0.001
Age (year)					0.010 (0.008, 0.012)	<0.001
Smoking History: yes vs. no					−0.014 (−0.075, 0.047)	0.6463

## Data Availability

Due to patient privacy concerns, the data from this study can be accessed by contacting the corresponding authors with a reasonable request.

## Supplementary Materials

The following supporting information can be downloaded at https://www.mdpi.com/article/10.3390/ijms26094443/s1.

## Abbreviations

The following abbreviations are used in this manuscript:

ACEIs	angiotensin-converting enzyme inhibitors
ARBs	angiotensin receptor blockers
AT	adipose tissue
BMI	body mass index
CVD	cardiovascular disease
HbA1c	glycated hemoglobin
HDL-C	high-density lipoprotein cholesterol
HOMA-IR	homeostasis model assessment of insulin resistance
HOMA B	homeostasis model assessment of beta-cell function
IL-1β	interleukin-1 beta
IL-6	interleukin-6
IL-8	interleukin-8
IS	insulin sensitivity
LDL-C	low-density lipoprotein cholesterol
MHNW	Metabolically Healthy Normal Weight
MHO	Metabolically Healthy Obese
MUNHNW	Metabolically Unhealthy Normal Weight
MUHO	Metabolically Unhealthy Obese
NSAIDs	non-steroidal anti-inflammatory drugs
TNF-α	tumor necrosis factor-alpha
TZDs	thiazolidinediones
