# Supplementary material for: The Association of Resistin with Metabolic Health and Obesity in a Mexican-American Population
Source: Int J Mol Sci. 2025 May 7;26(9):4443. doi: 10.3390/ijms26094443 (PMC12072545; doi:10.3390/ijms26094443)
Supplement: Supplementary file 1 [file ijms-26-04443-s001.zip › ijms-3565150-supplementary.pdf]

Adipokine in Metabolic Health/Obesity Group	Ln Resistin – Model 4		Ln Leptin – Model 4		Ln Adiponectin- Model 4	
	Mean Difference (95% CI)	*p*-value	Mean Difference (95% CI)	*p*-value	Mean Difference (95% CI)	*p*-value
**Statins**						
**Metabolic Health/Obesity Group**		0.8299		<.0001		<.0001
MHO vs. MHNW	0.084 (-0.151, 0.319)	0.4820	1.030 (0.902, 1.157)	<.0001	-0.177 (-0.275, -0.078)	0.0004
MUHNW vs. MHNW	0.035 (-0.153, 0.224)	0.7147	0.289 (0.187, 0.391)	<.0001	-0.311 (-0.390, -0.232)	<.0001
MUHO vs. MHNW	0.071 (-0.097, 0.238)	0.4089	1.028 (0.937, 1.119)	<.0001	-0.448 (-0.518, -0.378)	<.0001
MUHNW vs. MHO	-0.049 (-0.289, 0.191)	0.6881	-0.741 (-0.871, -0.611)	<.0001	-0.135 (-0.235, -0.034)	0.0086
MUHO vs. MHO	-0.014 (-0.238, 0.210)	0.9049	-0.002 (-0.123, 0.120)	0.9807	-0.271 (-0.365, -0.178)	<.0001
MUHO vs. MUHNW	0.035 (-0.128, 0.199)	0.6713	0.739 (0.650, 0.828)	<.0001	-0.137 (-0.205, -0.068)	<.0001
IL-1Beta_pg/mL	-0.010 (-0.020, 0.001)	0.0700	0.005 (-0.001, 0.010)	0.1181	-0.004 (-0.008, 0.001)	0.1044
IL-6_pg/mL	-0.0001 (-0.001, 0.0001)	0.8540	-0.0002 (-0.0005, 0.0001)	0.2785	0.0002 (0.0001, 0.0005)	0.0428
TNF-Alfa_pg/mL	-0.011 (-0.020, -0002)	0.0205	0.005 (0.0001, 0.010)	0.0427	0.0001 (-0.004, 0.004)	0.9201
IL-8_pg/mL	0.077 (0.063, 0.091)	<.0001	0.006 (-0.002, 0.013)	0.1367	0.010 (0.004, 0.015)	0.0010
Sex: Male vs. Female	0.038 (-0.102, 0.177)	0.5954	-1.128 (-1.203, -1.052)	<.0001	-0.258 (-0.317, -0.200)	<.0001
Age (year)	-0.003 (-0.008, 0.002)	0.2430	-0.001 (-0.004, 0.001)	0.3944	0.010 (0.008, 0.012)	<.0001
Smoking History: yes vs. no	0.116 (-0.029, 0.261)	0.1180	-0.065 (-0.144, 0.013)	0.1043	-0.014 (-0.075, 0.047)	0.6605
**Statins: yes vs. no**	-0.002 (-0.187, 0.183)	0.9840	0.070 (-0.030, 0.170)	0.1720	0.026 (-0.051, 0.104)	0.5045
**TZDs**						
**Metabolic Health/Obesity Group**		0.7875		<0.0001		<0.0001
MHO vs. MHNW	0.081 (-0.153, 0.315)	0.4974	1.026 (0.898, 1.153)	<.0001	-0.177 (-0.276, -0.079)	0.0004
MUHNW vs. MHNW	0.049 (-0.139, 0.237)	0.6110	0.293 (0.190, 0.395)	<.0001	-0.313 (-0.392, -0.234)	<.0001
MUHO vs. MHNW	0.083 (-0.083, 0.250)	0.3280	1.035 (0.944, 1.126)	<.0001	-0.488 (-0.518, -0.378)	<.0001
MUHNW vs. MHO	-0.032 (-0.271, 0.206)	0.7912	-0.733 (-0.863, -0.603)	<.0001	-0.135 (-0.236, -0.035)	0.0082
MUHO vs. MHO	0.002 (-0.220, 0.224)	0.9851	0.009 (-0.112, 0.130)	0.8799	-0.271 (-0.364, -0.177)	<.0001
MUHO vs. MUHNW	0.034 (-0.129, 0.197)	0.6795	0.742 (0.654, 0.831)	<.0001	-0.135 (-0.204, -0.067)	0.0001
IL-1Beta_pg/mL	-0.010 (-0.021, 0.0001)	0.0600	0.005 (-0.001, 0.010)	0.1195	-0.004 (-0.008, 0.001)	0.1104
IL-6_pg/mL	-0.0001 (-0.001, 0.001)	0.9060	-0.0002 (-0.0005, 0.0001)	0.3045	0.0002 (0.0001, 0.0005)	0.0432
TNF-Alfa_pg/mL	-0.011 (-0.020, -0.001)	0.0232	0.005 (0.0001, 0.010)	0.0447	0.0001 (-0.004, 0.004)	0.9493
IL-8_pg/mL	0.077 (0.063, 0.091)	<.0001	0.006 (-0.002, 0.013)	0.1402	0.010 (0.004, 0.015)	0.0010
Sex: Male vs. Female	0.053 (-0.086, 0.192)	0.4564	-1.124 (-1.200, -1.048)	<.0001	-0.260 (-0.319, -0.202)	<.0001
Age (year)	-0.002 (-0.007, 0.002)	0.3588	-0.0001 (-0.003, 0.002)	0.7516	0.010 (0.008, 0.012)	<.0001
Smoking History: yes vs. no	0.114 (-0.031, 0.259)	0.1233	-0.065 (-0.144, 0.014)	0.1056	-0.013 (-0.074, 0.048)	0.6726

**TZDs: yes vs. no**	-0.862 (-1.346, -0.378)	0.0005	-0.083 (-0.347, 0.181)	0.5383	0.150 (-0.053, 0.354)	0.1480
**Incretin Mimetics**						
**Metabolic Health/Obesity Group**		0.8422		<0.0001		<0.0001
MHO vs. MHNW	0.085 (-0.150, 0.320)	0.4788	1.026 (0.898, 1.153)	<.0001	-0.179 (-0.277, -0.080)	0.0004
MUHNW vs. MHNW	0.033 (-0.156, 0.222)	0.7306	0.293 (0.190, 0.395)	<.0001	-0.309 (-0.388, -0.230)	<.0001
MUHO vs. MHNW	0.067 (-0.101, 0.234)	0.4341	1.036 (0.945, 1.127)	<.0001	-0.443 (-0.513, -0.372)	<.0001
MUHNW vs. MHO	-0.052 (-0.291, 0.188)	0.6720	-0.733 (-0.863, -0.603)	<.0001	-0.130 (-0.230, -0.030)	0.0110
MUHO vs. MHO	-0.018 (-0.241, 0.205)	0.8743	0.011 (-0.111, 0.132)	0.8637	-0.264 (-0.357, -0.171)	<.0001
MUHO vs. MUHNW	0.034 (-0.130, 0.197)	0.6864	0.744 (0.655, 0.832)	<.0001	-0.134 (-0.203, -0.065)	0.0001
IL-1Beta_pg/mL	-0.010 (-0.020, 0.001)	0.0718	0.005 (-0.001, 0.010)	0.1204	-0.004 (-0.008, 0.001)	0.0994
IL-6_pg/mL	-0.0001 (-0.001, 0.0001)	0.8561	-0.0002 (-0.0005, 0.0001)	0.2968	0.0002 (0.0001, 0.0005)	0.0412
TNF-Alfa_pg/mL	-0.011 (-0.020, -0.002)	0.0207	0.005 (0.0001, 0.010)	0.0462	0.0001 (-0.004, 0.004)	0.9375
IL-8_pg/mL	0.077 (0.063, 0.091)	<.0001	0.006 (-0.002, 0.013)	0.1337	0.010 (0.004, 0.016)	0.0009
Sex: Male vs. Female	0.037 (-0.102, 0.176)	0.6023	-1.125 (-.201, -1.049)	<.0001	-0.257 (-0.315, -0.199)	<.0001
Age (year)	-0.003 (-0.008, 0.001)	0.1876	-0.0001 (-0.003, 0.002)	0.7679	0.010 (0.008, 0.012)	<.0001
Smoking History: yes vs. no	0.115 (-0.030, 0.260)	0.1212	-0.064 (-0.143, 0.015)	0.1104	-0.013 (-0.073, 0.048)	0.6857
**Incretin Mimetics: yes vs. no**	0.107 (-0.271, 0.485)	0.5800	-0.073 (-0.278, 0.132)	0.4833	-0.102 (-0.260, 0.057)	0.2084
**ACE-Is/ARBs**						
**Metabolic Health/Obesity Group**		0.8094		<0.0001		<0.0001
MHO vs. MHNW	0.084 (-0.150, 0.319)	0.4813	1.026 (0.899, 1.154)	<.0001	-0.178 (-0.276, -0.080)	0.0004
MUHNW vs. MHNW	0.040 (-0.151, 0.230)	0.6821	0.295 (0.192, 0.399)	<.0001	-0.314 (-0.394, -0.235)	<.0001
MUHO vs. MHNW	0.078 (-0.094, 0.249)	0.3756	1.040 (0.947, 1.133)	<.0001	-0.452 (-0.524, -0.380)	<.0001
MUHNW vs. MHO	-0.045 (-0.285, 0.196)	0.7164	-0.731 (-0.861, -0.600)	<.0001	-0.136 (-0.237, -0.035)	0.0081
MUHO vs. MHO	-0.007 (-0.233, 0.219)	0.9531	0.014 (-0.109, 0.137)	0.8244	-0.274 (-0.369, -0.179)	<.0001
MUHO vs. MUHNW	0.038 (-0.126, 0.202)	0.6515	0.745 (0.655, 0.834)	<.0001	-0.138 (-0.206, -0.069)	<.0001
IL-1Beta_pg/mL	-0.010 (-0.020, 0.001)	0.0707	0.005 (-0.001, 0.010)	0.1152	-0.004 (-0.008, 0.001)	0.1030
IL-6_pg/mL	-0.0001 (-0.001, 0.001)	0.8619	-0.0002 (-0.0005, 0.0001)	0.3071	0.0002 (0.0001, 0.0005)	0.0427
TNF-Alfa_pg/mL	-0.011 (-0.020, -0.002)	0.0203	0.005 (0.0001, 0.010)	0.0464	0.0001 (-0.004, 0.004)	0.9249
IL-8_pg/mL	0.077 (0.063, 0.091)	<.0001	0.006 (-0.002, 0.013)	0.1354	0.010 (0.0041, 0.015)	0.0011
Sex: Male vs. Female	0.039 (-0.101, 0.178)	0.5849	-1.124 (-1.200, -1.049)	<.0001	-0.259 (-0.317, -0.200)	<.0001
Age (year)	-0.003 (-0.007, 0.002)	0.2982	-0.0001 (-0.003, 0.002)	0.8851	0.010 (0.008, 0.012)	<.0001
Smoking History: yes vs. no	0.114 (-0.032, 0.260)	0.1248	-0.066 (-0.146, 0.013	0.0994	-0.012 (-0.073, 0.049)	0.7009
**ACE-Is/ARBs: yes vs. no**	-0.032 (-0.208, 0.144)	0.7243	-0.027 (-0.123, 0.068)	0.5777	0.027 (-0.047, 0.100)	0.4782
**Fibrates**						

**Metabolic Health/Obesity Group**		0.8535		<0.0001		<0.0001
MHO vs. MHNW	0.083 (-0.152, 0.318)	0.4899	1.029 (0.902, 1.156)	<.0001	-+0.176 (-0.275, -0.078)	0.0004
MUHNW vs. MHNW	0.031 (-0.157, 0.220)	0.7449	0.298 (0.195, 0.400)	<.0001	-0.307 (-0.386, -0.228)	<.0001
MUHO vs. MHNW	0.065 (-0.103, 0.232)	0.4502	1.044 (0.953, 1.134)	<.0001	-0.440 (-0.510, -0.370)	<.0001
MUHNW vs. MHO	-0.051 (-0.291, 0.188)	0.6737	-0.731 (-0.861, -0.602)	<.0001	-0.130 (-0.230, -0.030)	0.0108
MUHO vs. MHO	-0.018 (-0.241, 0.205	0.8730	0.015 (-0.106, 0.136)	0.8094	-0.264 (-0.357, -0.170)	<.0001
MUHO vs. MUHNW	0.033 (-0.130, 0.197)	0.6906	0.746 (0.657, 0.835)	<.0001	-0.133 (-0.202, -0.065)	0.0001
IL-1Beta_pg/mL	-0.010 (-0.021, 0.001)	0.6666	0.005 (-0.001, 0.011)	0.1013	-0.004 (-0.008, 0.001)	0.1162
IL-6_pg/mL	-0.0010 (-0.001, 0.001)	0.8632	-0.0002 (-0.0005, 0.0001)	0.2804	0.0002 (0.0001, 0.00005)	0.0432
TNF-Alfa_pg/mL	-0.011 (-0.020, -0.002)	0.0213	0.005 (-0.0001, 0.010)	0.0500	0.0001 (-0.004, 0.004)	0.9569
IL-8_pg/mL	0.077 (0.063, 0.091)	<.0001	0.006 (-0.0001, 0.0013)	0.1170	0.010 (0.004, 0.016)	0.0008
Sex: Male vs. Female	0.033 (-0.107, 0.173)	0.6424	-1.118 (-1.193, -1.042)	<.0001	-0.253 (-0.312, -0.195)	<.0001
Age (year)	-0.003 (-0.008, 0.001)	0.1822	-0.0001 (-0.003, 0.002)	0.8847	0.010 (0.008, 0.012)	<.0001
Smoking History: yes vs. no	0.120 (-0.026, 0.265)	0.1065	-0.071 (-0.150, 0.007)	0.0755	-0.017 (-0.078, 0.044)	0.5767
**Fibrates: yes vs. no**	0.173 (-0.212, 0.558)	0.3785	-0.287 (-0.495, -0.078)	0.0071	-0.169 (-0.330, -0.008)	0.0402
**NSAIDs**						
**Metabolic Health/Obesity Group**		0.8433		<0.0001		<0.0001
MHO vs. MHNW	0.086 (-0.149, 0.321)	0.4720	1.026 (0.899, 1.154)	<.0001	-0.177 (-0.276, -0.079)	0.0004
MUHNW vs. MHNW	0.032 (-0.157, 0.221)	0.7388	0.291 (0.189, 0.394)	<.0001	-0.311 (0.391, -0.232)	<.0001
MUHO vs. MHNW	0.066 (-0.102, 0.233)	0.4433	1.033 (0.943, 1.124)	<.0001	-0.448 (-0.518, -0.377)	<.0001
MUHNW vs. MHO	-0.054 (-0.294, 0.186)	0.6582	-0.735 (-0.865, -0.605)	<.0001	-0.134 (-0.234, -0.034)	0.0089
MUHO vs. MHO	-0.021 (-0.244, 0.203)	0.8563	0.007 (-0.114, 0.129)	0.9060	-0.270 (-0.364, -0.177)	<.0001
MUHO vs. MUHNW	0.033 (-0.130, 0.197)	0.6888	0.742 (0.654, 0.831)	<.0001	-0.136 (-0.205, -0.068)	<.0001
IL-1Beta_pg/mL	-0.010 (-0.020, 0.001)	0.0730	0.005 (-0.001, 0.010)	0.1165	-0.004 (-0.008, 0.001)	0.1085
IL-6_pg/mL	-0.0001 (-0.001, 0.0001)	0.8349	-0.0002 (-0.0005, 0.0001)	0.2977	0.0002 (0.0001, 0.0005)	0.0425
TNF-Alfa_pg/mL	-0.011 (-0.020, -0.002)	0.0211	0.005 (0.0001, 0010)	0.0456	0.0001 (-0.004, 0.004)	0.9240
IL-8_pg/mL	0.077 (0.063, 0.091)	<.0001	0.006 (-0.002, 0.013)	0.1409	0.010 (0.004, 0.015)	0.0011
Sex: Male vs. Female	0.036 (-0.103, 0.176)	0.6079	-1.126 (-1.201, -1.050)	<.0001	-0.258 (-0.316, -0.200)	<.0001
Age (year)	-0.003 (-0.008, 0.001)	0.1651	-0.0001 (-0.003, 0.002)	0.6960	0.010 (0.008, 0.012)	<.0001
Smoking History: yes vs. no	0.117 (-0.029, 0.262)	0.1156	-0.065 (-0.144, 0.014)	0.1071	-0.013 (-0.074, 0.048)	0.6703
**NSAIDs: yes vs. no**	0.068 (-0.114, 0.250)	0.4612	0.005 (-0.094, 0.104)	0.9225	0.023 (-0.053, 0.100)	0.5479

Supplementary Table S1. Associations Between Metabolic Health/Obesity Status and Log-Trans�ormed Adipkines based on Multivariable (Adjusted) linear regression Model 4.
